# Cancer-associated thoracic aorta arterial thrombosis: case report and review of the literature

**DOI:** 10.3389/fcvm.2025.1480310

**Published:** 2025-01-29

**Authors:** Miguel Borregón, María Valero, Asia Ferrández, Álvaro Muñoz, Carmen Roque, Javier-David Benítez-Fuentes

**Affiliations:** ^1^ Department of Medical Oncology, Hospital General Universitario de Elche, FISABIO, Alicante, Spain; ^2^ Department of Radiology, Hospital General Universitario de Elche, FISABIO, Alicante, Spain

**Keywords:** cancer-associated thrombosis (CAT), venous thromboembolism (VTE), arterial thromboembolism (ATE), low molecular weight heparin (LMWH), small cell lung carcinoma (SCLC) and cisplatin

## Abstract

**Background:**

Arterial thrombosis is an uncommon complication in cancer patients, often overshadowed by venous thromboembolic events. Its occurrence in patients with solid tumors on active antineoplastic treatment poses a significant clinical challenge.

**Key clinical question:**

Given the lack of consensus on the optimal therapy for arterial thrombosis in cancer patients, the best practices for managing an aortic thrombus, and the benefit of low molecular weight heparin (LMWH) must be reviewed.

**Clinical approach:**

We present the case of a 70-year-old female with stage IVA lung adenocarcinoma who developed an aortic thrombus during chemo-immunotherapy. The thrombus was successfully treated with LMWH, avoiding further complications, and allowing for the continuation of her cancer therapy.

**Conclusions:**

This case highlights the importance of early detection and management of arterial thrombus in cancer patients. LMWH proved effective in resolving the thrombus, underscoring its role in managing such complications.

## Introduction

Cancer-associated thrombosis (CAT) is a well-recognized complication in cancer patients, contributing significantly to morbidity and mortality in this population. Thrombosis in cancer patients is multifactorial, involving tumor-related factors, patient characteristics, and treatment modalities (1). While venous thromboembolism (VTE) is the most common type of CAT, arterial thromboembolism (ATE), although less frequent, poses a critical risk, leading to severe cardiovascular events, including myocardial infarction, cerebrovascular events, and peripheral artery disease (2).

The pathophysiology of ATE in cancer involves complex interactions between pro-thrombotic states induced by malignancy, endothelial damage, and hypercoagulability exacerbated by cancer treatments (3). Different epidemiological studies indicate that cancer patients have an elevated risk of ATE compared to the general population (4). However, information related to its management is scarce (3).

The case discussed herein involves a 70-year-old woman with a history of limited stage small cell lung carcinoma (SCLC) treated with radical chemo-radiotherapy in 2008 and a recent diagnosis of stage IVA adenocarcinoma of the lung in December 2023. Her current treatment includes a combination of chemotherapy and immunotherapy (maintenance pemetrexed and pembrolizumab). After a partial response to treatment, the patient developed an arterial thrombus in the ascending aorta. This complication highlights the potential risk for serious thrombotic complications in cancer patients, the importance of cardiovascular monitoring and prompt treatment of CAT in oncology patients.

## Case description

A timeline of the key events in this case is presented in Table 1. A 70-year-old Caucasian woman with a significant past medical history presented with asymptomatic aortic thrombosis. Her personal history includes a former smoking habit (35 pack-years, ceased in 2008), hypertension, dyslipidemia, osteoporosis under oral zoledronic acid treatment, and actinic keratosis on the nose. Her surgical history includes resection of breast fibroadenomas and a basal cell carcinoma excision from the right shoulder in 2006. She had no previous medical history of thrombosis.

**Table 1:** Timeline of key events.

July 2008	Second semester 2008	January 2009	December 2023	First trimester 2024	March 2023	Second trimester 2024	May 31, 2024	June 5, 2024	June 14, 2024
Limited stage small cell lung carcinoma	Cisplatin + etoposide + concurrent thoracic radiotherapy	Prophylactic cranial irradiation	Stage IVA adenocarcinoma of the lung	Carboplatin + pemetrexed + pembrolizumab	Partial response	Pemetrexed + pembrolizumab maintenance	Ascending thoracic aorta thrombus	LMWH 1 mg kg	Thrombus is resolved

The patient's oncological history is notable for two metachronous neoplasms. In July 2008, she was diagnosed with limited stage SCLC of the right lung with a 9.5 cm right anterior mediastinal lymph node mass, which compressed the superior cava vein, a 4.5 cm subpleural right lung mass, and enlarged subcarinal nodes. She underwent five cycles chemotherapy with cisplatin (80 mg/m^2^ IV infusion) and etoposide (100 mg/m^2^ IV infusion) every 3 weeks and concurrent radiotherapy 50.4 Gy in 30 sessions on the pulmonary mass and the affected lymph nodes. In January 2009 she received prophylactic cranial irradiation (PCI), 30 Gy in 10 sessions. She achieved a major partial response and was followed up until March 2020 with no evidence of progression, when she was discharged from oncology following an unremarkable FDG-PET scan.

In December 2023, she was diagnosed with stage IVA (cT2b cN1 cM1a) adenocarcinoma of the lung, characterized by a 5 cm necrotic mass in inferior right lobe (SuvMax 10.9), a small node located in lingula (SuvMax 4.5), and two right hilar adenopathies (SuvMax 6.6). The biopsy sample was negative for PD-L1 expression, and driver mutations. She started first-line treatment with carboplatin (AUC 5 IV infusion), pemetrexed (500 mg/m² IV infusion) and pembrolizumab (200 mg IV infusion) every 3 weeks. After four cycles, an FDG-PET scan in March 2024 showed a partial response, and maintenance therapy with pemetrexed (500 mg/m² IV infusion) and pembrolizumab (200 mg IV infusion) was initiated.

**Figure 1:**
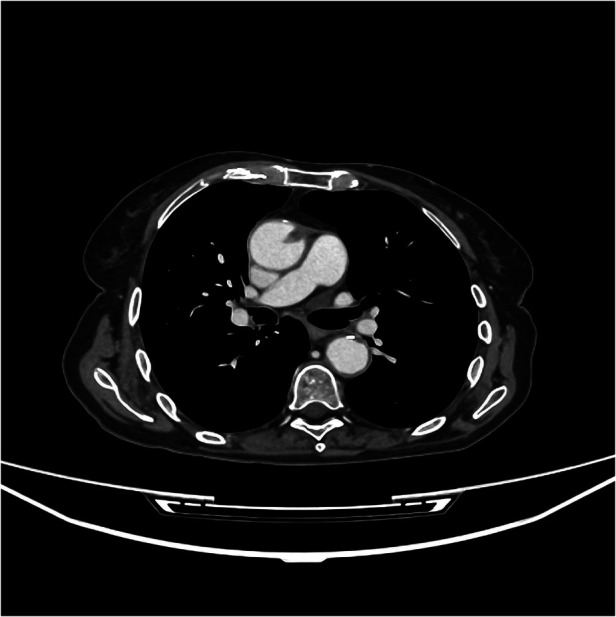
Transversal CT-scan plane showing the 8 mm thrombus in the ascending aorta.

**Figure 2:**
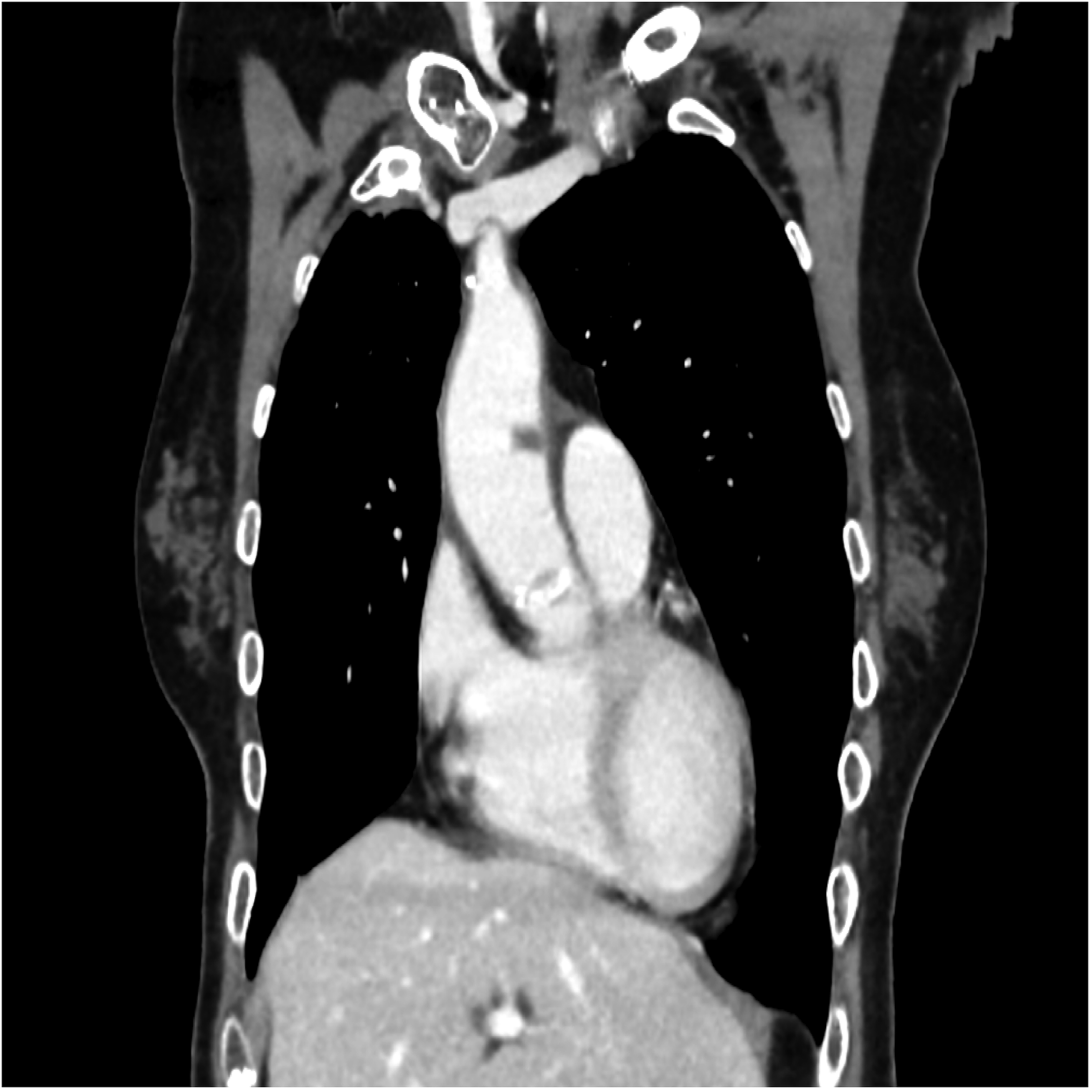
Coronal CT-scan plane showing the 8 mm thrombus in the ascending aorta.

**Figure 3:**
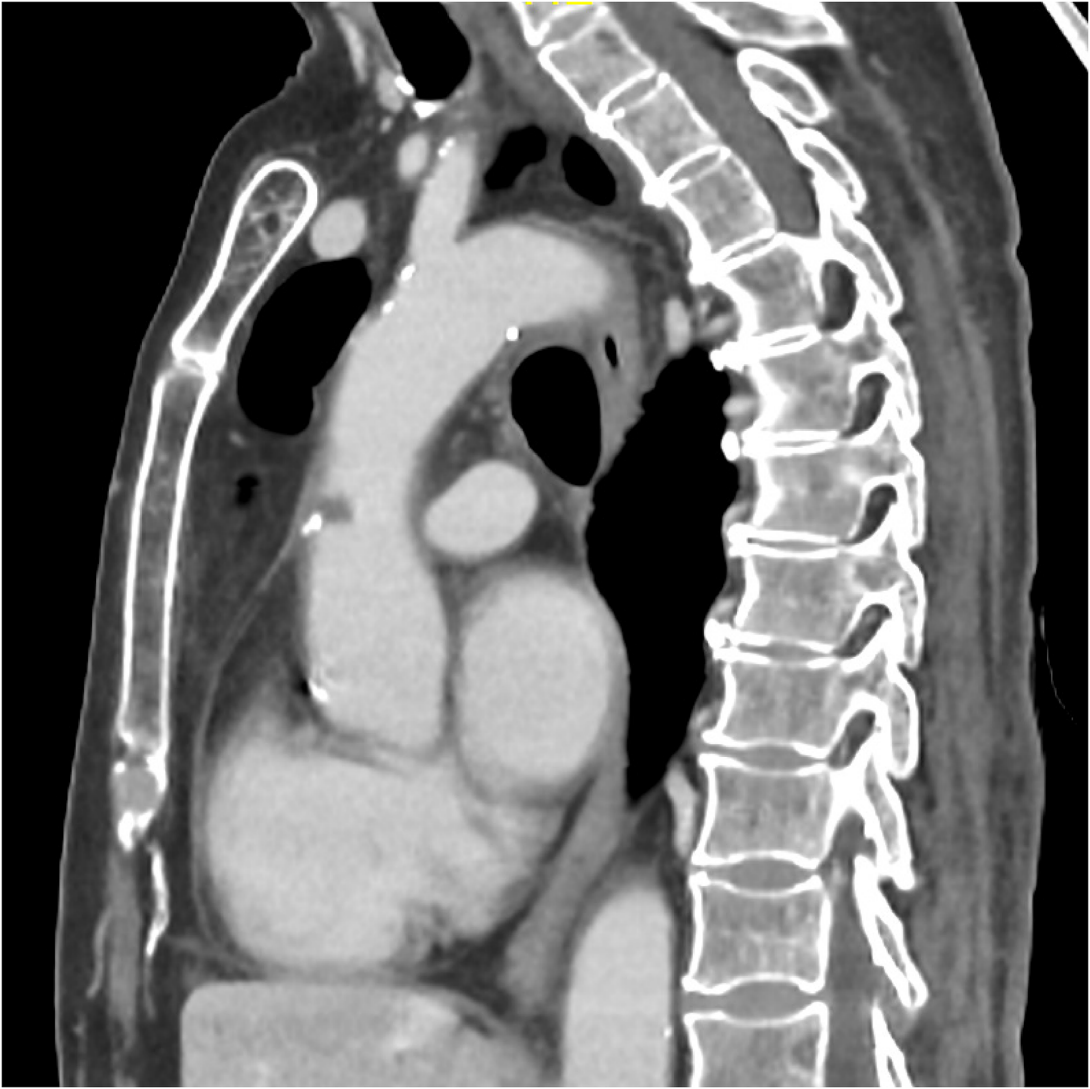
Sagittal CT-scan plane showing the 8 mm thrombus in the ascending aorta.

On June 5, 2024, during a routine follow-up visit, she was diagnosed with an asymptomatic ascending aorta arterial thrombus after completing four cycles of pemetrexed and pembrolizumab maintenance therapy. The thoraco-abdominal CT scan for her tumor evaluation, carried out on May 31, 2024, revealed a new hypodense nodular image adhered to the anterior wall of the ascending thoracic aorta, consistent with an 8 mm thrombus (Figures 1–3)*.* The CT scan also noted chronic post-radiation changes in the right lung and a stable subpleural necrotic mass in the right lower lobe, with no evidence of new mediastinal adenopathy or pleural/pericardial effusion.

As risk factors for the thrombus development, besides her oncology history, the patient had also been diagnosed 2 weeks before with a fracture of the sacrum. It was related to her osteoporosis and an accidental fall at home days before. Magnetic resonance performed on May 22, 2024, described a vertical and bilateral sacral ala fracture, with marrow edema, with no tumoral component. She was experiencing severe pain and was prescribed transdermal fentanyl.

Given the thrombus diagnosis, the oncology team suspended the patient's maintenance therapy and consulted the cardiology team, that evaluated the patient on the same day. Cardiology evaluation found no chest pain, signs of heart failure, syncope, palpitations, or neurological symptoms. An echocardiogram revealed a non-dilated and non-hypertrophic left ventricle with preserved ejection fraction and no segmental wall motion abnormalities. There was moderate bi-atrial dilation, a normal-sized aortic root (31 mm) and ascending aorta (36 mm) without alterations. A small nodule was visualized where the aortic thrombus was located in the CT. No pericardial effusion or intracavitary thrombus were seen. For therapeutic decision-making, we conducted a thorough review of the literature regarding ATE treatment in cancer patients. In most published case reports, asymptomatic ATE has been managed with LMWH. Therefore, we decided to start enoxaparin 1 mg/kg every 12 hours.

**Figure 4:**
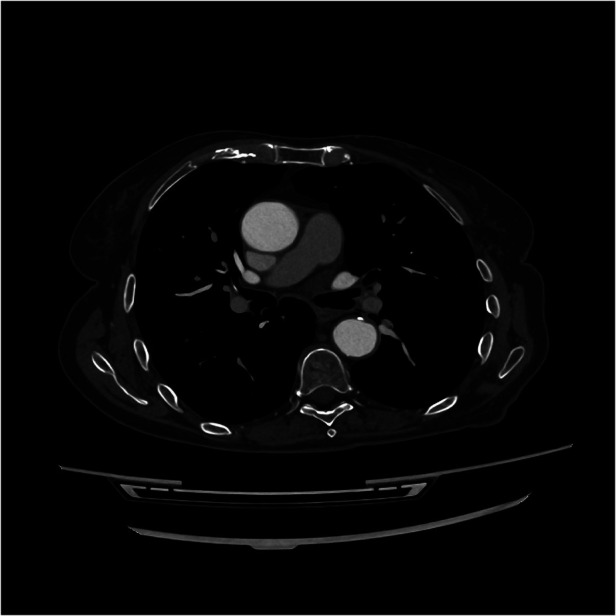
Transversal CT-scan plane showing the resolution of the 8 mm thrombus in the ascending aorta.

**Figure 5:**
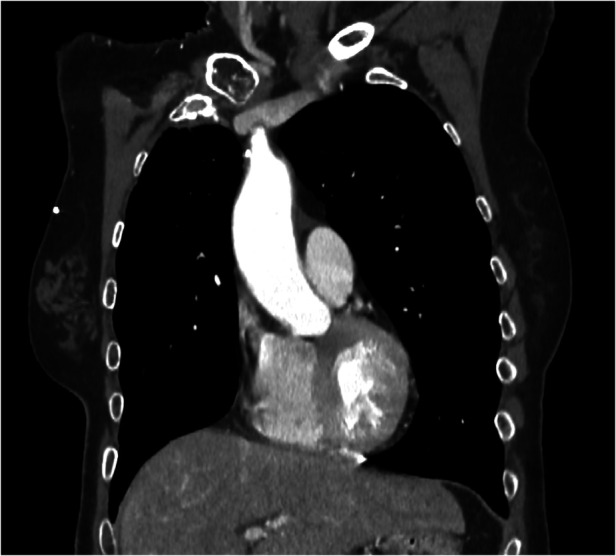
Coronal CT-scan plane showing the resolution of the 8 mm thrombus in the ascending aorta.

**Figure 6:**
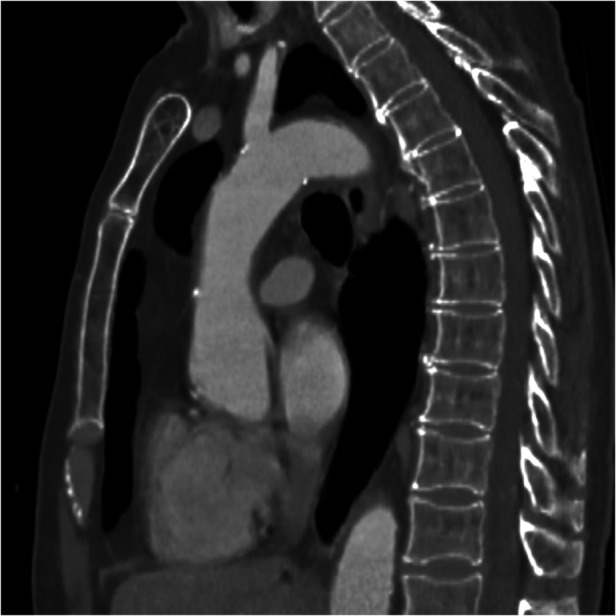
Sagittal CT-scan plane showing the resolution of the 8 mm thrombus in the ascending aorta.

Two weeks later, on June 14, 2024, a follow-up CT scan of the chest to monitor the aortic thrombus showed its complete resolution (Figures 4–6)*.* Due to the patient's preference to avoid twice-daily injections, her anticoagulation therapy was changed to bemiparin 115 IU/Kg every 24 hours and she resumed her treatment with pemetrexed and pembrolizumab. The patient reported no new symptoms and developed no complications related to the anticoagulation therapy. At the three-month follow-up, she remained asymptomatic.

## Discussion

This case highlights the importance of vigilant cardiovascular monitoring in cancer patients, especially those undergoing active treatment, due to the heightened risk of thrombotic events. Early identification and prompt management of arterial thrombosis are critical in improving outcomes in this vulnerable population. Our patient was exposed to several well-known risk factors for thrombotic events, including her past SCLC, her current adenocarcinoma of the lung, the chemotherapy, thoracic radiotherapy and PCI in 2008, her current treatment with chemotherapy and immunotherapy, and her recent sacrum fracture. However, our patient did not exhibit the typical clinical presentation of VTE. Instead, she developed an ATE in the ascending aorta, which is particularly challenging to treat due to its critical location and associated compromise. The literature on managing ATEs is scarce, underscoring the need for individualised assessment and management in each case.

Cancer patients experience an increased risk of thromboembolic events due to a hypercoagulable state induced by the malignancy itself, as well as by the therapeutic interventions they undergo. It has been reported that the risk of VTE increases four- to six-fold in cancer patients receiving chemotherapy (5, 6), and especially in the case of ATE it can lead to catastrophic outcomes (7). Risk factors for ATE in the general population are severe atherosclerosis, aortic aneurysm, cardiovascular surgery, uncontrolled diabetes mellitus and polytrauma (8–10). Some ATE risk factors documented in cancer patients are: the first month after diagnosis of cancer, hypertension, smoking, pre-existing cardiovascular diseases, age over 65 years, previous history of ATE, distant metastasis and chemotherapy (11). While ATE in cancer patients is less common than VTE, both conditions share overlapping pathophysiological mechanisms (12). Recognized cancer-related mechanisms contributing to these complications include tumour cell-induced activation of coagulation pathways, a decrease in anticoagulant molecules, structural or functional changes in the vascular wall, endothelial damage, platelet activation, alterations in the fibrinolytic system, and the release of cytokines such as vascular endothelial growth factor (VEGF), tumour necrosis factor (TNF)-α, and interleukin-1β (13–18).

Antineoplastic agents, including hormone therapy, chemotherapy, and immunotherapy play an additional role, further exacerbating the risk by inducing endothelial injury and enhancing procoagulant activity (19, 20). Radiotherapy has also been suggested as a risk factor for VTE development. In a prospective study focused on curative intent radiotherapy, Daguenet et al. found an incidence rate of VTE 6 months post-radiotherapy of 2% (21).

Cisplatin and carboplatin are some of the frequently used chemotherapy drugs associated with ATE. It has been speculated that cisplatin may enhance tissue factor activity and platelet activation and elevate the von Willebrand factor, which can cause endothelial injury and potentiate arterial thrombosis. Dehydration and hypomagnesemia, are factors related to cisplatin treatment that might also contribute to thrombosis (22–25).

Some clinical cases specifically explore the relationship between cisplatin and ATE. Table 2 summarizes these cases. Chin et al. described the case of one patient with limited stage SCLC treated with cisplatin and etoposide, who was diagnosed with an aortic arch thrombus and its improvement when started on LMWH (26). Weijl et al. studied the incidence of thrombosis in 179 germ cell cancer patients treated with cisplatin and bleomycin, finding it to be approximately 8.4% (18). Of these events, three (16.7%) were arterial events, including two cerebral ischemic strokes and one lower limb arterial thrombosis. Numico et al. found in 108 stage III-IV NSCLC patients treated with cisplatin and gemcitabine chemotherapy a VTE incidence of approximately 17.6% (27). Of these events, ten were arterial events (2 myocardial infarctions, 7 lower limb arterial thrombosis, and 1 cerebral ischemic stroke). Dieckmann et al. reported the case of a man with advanced seminoma receiving cisplatin, etoposide and bleomycin chemotherapy who developed thrombotic deposits in the descending thoracic aorta and in the infrarenal abdominal aorta respectively. He was placed on anticoagulant therapy and 6 months after completion of chemotherapy, thrombotic deposits had completely resolved (28). Apiyasawat et al. reported the case of a patient that developed a thrombus in the distal arch of the aorta when she was receiving radiotherapy, cisplatin and 5-fluorouracil for stage IIB cervical cancer (29). Numerous vascular emboli were occluding her distal common femoral artery. The patient underwent successful surgical thrombectomy. Ito et al. described a patient suffering a thrombosis in the descending arch of the thoracic aorta and the infrarenal abdominal aorta during cisplatin-based chemotherapy for stage IV gastric cancer that was successfully treated with intravenous heparin and warfarin (30). Morlese et al. described a case of spontaneous arterial thrombosis in a 42-year-old male with localised oesophageal adenocarcinoma the day after receiving his first cycle of preoperative cisplatin-5-fluorouracil chemotherapy (31). It was identified a large thrombus in the abdominal aorta, beginning in the mid-aorta, just superior to the origin of the renal arteries, and continuing distally into both common iliac vessels. The patient received a surgical embolectomy and bilateral lower limb fasciotomies, but she finally died 7 days later due to small bowel infarction. Moore et al. performed a large retrospective analysis of all patients treated with cisplatin-based chemotherapy at *Memorial Sloan-Kettering Cancer Center* in 2008. Among 932 patients, 169 (18.1%) experienced a VTE and/or an ATE event during cancer treatment or within 4 weeks after finishing it. Most of them suffered VTE, but arterial thrombosis alone was found in 8.3% of these patients, and co-occurrence of ATE and VTE in 3.0% (32).

**Table 2:** Clinical cases exploring the relationship between cisplatin and arterial thromboembolism (ATE).

Author	Publication	Cancer type	Chemotherapy	Site of ATE	Timing of ATE	Outcomes
Chin et al. (26)	2010	1 patient with limited stage SCLC	Cisplatin + etoposide	Aortic arch thrombus	First restaging scan	Improvement on LMWH
Weijl et al. (18)	2000	179 patients with germ cell cancer	Cisplatin + bleomycin-based CT	2 strokes, 1 lower limb ATE	Between the start and 6 weeks after CT	Incidence of ATE 1,7%
Numico et al. (27)	2005	108 patients with stage III-IV NSCLC	Cisplatin + gemcitabine	2 myocardial infarctions, 7 lower limb ATEs, 1 stroke	Between 4 days and 234 days after the start of CT	Incidence of ATE 9,2%
Dieckmann et al. (28)	2009	1 patient with advanced seminoma	Cisplatin + etoposide + bleomycin	Descending thoracic aorta and infrarenal aorta	First restaging scan after 2 cycles of CT	Improvement on LMWH, AAS and warfarin
Apiyasawat et al. (29)	2003	1 patient with stage IIB cervical cancer	RT + cisplatin + 5-fluorouracil	Aortic distal arch and femoral artery emboli	The same day receiving CT	Successful surgical thrombectomy
Ito et al. (30)	2013	1 patient with stage IV gastric cancer	Cisplatin-based CT	Descending thoracic aorta and infrarenal abdominal aorta	First restaging scan	Successfully treated with intravenous heparin and warfarin
Morlese et al. (31)	2007	1 patient with localized oesophageal adenocarcinoma	Preoperative cisplatin + 5- fluorouracil	Large thrombus in the abdominal aorta	The day after receiving his first cycle	Surgical embolectomy, finally died 7 days later
Moore et al. (32)	2011	All (932) patients with cisplatin-based CT in 2008	Cisplatin-based CT	14 patients with ATE, 5 patients with ATE + VTE	Between the start and 4 weeks after CT	Incidence of ATE 8,3%, and ATE + VTE 3%

AAS, acetylic salicylic acid; ATE, arterial thromboembolism; CT, chemotherapy; LMWH, low molecular weight heparin; SCLC, small cell lung carcinoma; NSCLC, non-small cell lung carcinoma; RT, radiotherapy; VTE, venous thromboembolism.

The patient we present in this case report has a history of thoracic radiotherapy for her previous SCLC, another notable risk factor for ATE development. A *post-hoc* analysis from the *COMPASS-CAT trial*, whose primary objective was to study a predictive model for VTE diagnosis in cancer, was focused on 361 patients with early, locally advanced, or metastatic breast, lung, colon, or ovarian cancer who received radiotherapy. At a median follow up of 6 months, 33 patients (9.1%) developed a VTE event. After applying a competing risk model, radiotherapy remained significantly associated with increased risk for VTE (HR 2.47, 95% CI: 1.47–4.12, *p* = 0.001) (33). Radiation injury has been demonstrated to cause acute and late alterations in the endothelium, favouring vascular events, as it has been studied in patients with central nervous system tumours presenting radionecrosis (34–36). Rishi et al. published the case of a 54-year-old male suffering extensive abdominal aortic thrombus along with involvement of left common iliac, saphenous-popliteal, and tibial arteries as well as moderate stenosis coronary arteries three days after receiving her first cycle of cisplatin-based chemotherapy given concurrently with radiotherapy for squamous cell carcinoma of the base of tongue (37).

Another risk factor for CAT development exhibited by the patient of our clinical case is her current exposure to pembrolizumab, as immune checkpoint inhibitors (ICI) have been associated with VTE and ATE risk in cancer patients (38–41). Moik et al. in the *Vienna General Hospital* conducted a retrospective study focused on patients treated with ICI. Melanoma, lung cancer and renal cancer were the most common types of cancer included. Nivolumab, pembrolizumab and ipilimumab were the most frequent ICI included. A total of 47.6% patients were also treated with radiation therapy. Cumulative incidences of VTE and ATE were 12.9% and 1.8% respectively (38). Based on the same hypothesis, Sussman et al. conducted a retrospective study of 228 patients with melanoma receiving ICI between 2015 and 2017 at the *Cleveland Clinic*. Fifty-one thrombotic events occurred in 47 patients (20.6%), including 37 VTE and 14 ATE (39). Roopkumar et al. conducted a retrospective cohort study including 1.686 patients who received ICI for a variety of malignancies to determine the incidence of VTE. VTE occurred in 404 patients (24%) and was associated with decreased overall survival [HR = 1.22 (95% CI 1.06–1.41), *p* < 0.008]. Patients developing VTE expressed significantly higher pretreatment levels of myeloid-derived suppressor cells, interleukin 8, and soluble vascular cell adhesion protein 1, postulated as drivers in VTE pathogenesis (40).

The information regarding the management of ATE in cancer patients is scarce and it should involve a multidisciplinary approach. Standard treatment modalities remain controversial. Systemic anticoagulation alone, aortic thromboendarterectomy, open-surgery for thrombus removal, or endovascular placement of stent grafts has been suggested (41–45). Goto et al. published the case of a 51-year-old woman with early esophageal cancer who presented an aortic mural thrombus. She was started on intravenous heparin and later changed to warfarin and three months later the thrombus had disappeared (46). Han et al*.* described a patient presenting ascending aortic thrombosis occurring 9 days after cisplatin-based chemotherapy for his lung cancer. He was successfully treated with LMWH and warfarin (47).

## Conclusion

Although less frequent than VTE, ATE in cancer patients requires prompt recognition and management due to its severe potential consequences. This case describes an ascending aortic ATE in a cancer patient successfully treated with LMWH. Anticoagulation with LMWH resolved the thrombus, demonstrating its efficacy in managing CAT. LMWH for ATE in cancer patients may prevent further vascular complications, and allow for the continuation of the antineoplastic treatment. We hypothesize that if there are no life-threatening symptoms and no contraindications, based on the scarce available evidence, multidisciplinary evaluation, anticoagulation with LMWH alone and a close follow-up could be a good option for cancer-related ATE.

## Data Availability

The original contributions presented in the study are included in the article/Supplementary Material, further inquiries can be directed to the corresponding author.
